# Celiac Artery Dissection in an HIV-Positive Patient With Cocaine Use: A Rare Vascular Emergency

**DOI:** 10.7759/cureus.92474

**Published:** 2025-09-16

**Authors:** Haashim Rahman, Abbas Merchant, Aaryan Patel, Adithya Nagendran, Constantino G Lambroussis

**Affiliations:** 1 Anesthesiology, Lake Erie College of Osteopathic Medicine, Erie, USA; 2 Anesthesia, Lake Erie College of Osteopathic Medicine, Erie, USA; 3 Physical Medicine and Rehabilitation, Lake Erie College of Osteopathic Medicine, Erie, USA; 4 Internal Medicine, Rochester Regional Health, Rochester, USA; 5 Osteopathic Medicine/Family Medicine, Lake Erie College of Osteopathic Medicine, Elmira, USA

**Keywords:** acute abdominal pain, cocaine-induced vasospasm, computed tomography angiography (cta), endothelial dysfunction, false lumen thrombosis, hiv-associated vasculopathy, non-operative management, spontaneous celiac artery dissection, visceral artery dissection

## Abstract

Celiac artery dissection is a rare vascular emergency that typically occurs in middle-aged men with risk factors like hypertension or atherosclerosis. We report a unique case of spontaneous celiac artery dissection in a patient with HIV infection and recent cocaine use. The patient presented to the emergency department with acute epigastric pain shortly after cocaine ingestion. Contrast-enhanced CT angiography confirmed an isolated dissection of the celiac artery without aortic involvement. Inpatient management was conservative, including strict blood pressure control, analgesia, and close monitoring, with input from cardiology and vascular surgery. The patient remained hemodynamically stable and was managed non-operatively, with plans for outpatient follow-up and risk-factor modification. This case highlights the potential synergistic role of cocaine’s hemodynamic effects and HIV-associated vasculopathy in precipitating visceral arterial dissection, and underscores the importance of considering visceral artery dissection in patients with atypical risk factors. The outcome in this case was favorable with medical management alone.

## Introduction

Spontaneous isolated celiac artery dissection (SICAD) is a rare but potentially life-threatening vascular event, second only to superior mesenteric artery dissection among visceral arterial dissections [1]. Since its initial description in 1959, fewer than 200 cases have been reported in the literature [2]. Most cases occur in middle-aged men, typically in the fifth decade of life, and are associated with traditional cardiovascular risk factors such as chronic hypertension, smoking, and atherosclerotic disease [2]. Additional predisposing conditions include connective tissue disorders (e.g., Marfan syndrome), blunt trauma, and pregnancy [3].

Despite these associations, a significant subset of SICAD cases occurs in younger patients with no identifiable predisposing factors, raising questions about underrecognized etiologies [3]. The pathogenesis of SICAD is not fully understood, but it is hypothesized to involve an underlying weakness in the arterial wall that becomes disrupted under acute hemodynamic stress, resulting in an intimal tear [2].

Two underappreciated but potentially synergistic risk factors for arterial dissection are HIV infection and cocaine use, both of which were present in our patient. HIV-associated vasculopathy is increasingly recognized in the setting of advanced HIV infection and is characterized by arterial inflammation, endothelial dysfunction, and accelerated atherosclerosis [4]. This vasculopathy may manifest as aneurysms, occlusive disease, or spontaneous arterial dissections across various vascular beds.

In adults with advanced HIV/AIDS, the prevalence of cerebral vasculitis-like vasculopathy has been reported as high as 23% [5]. Within documented HIV-associated cerebral vasculopathy cases, aneurysms are the most frequent manifestation, fusiform aneurysms account for ~75% of reported adult intracranial aneurysms, with saccular types comprising ~12.5% [5]. Occlusive disease, manifesting as stenotic or thrombotic lesions, has also been common, observed across multiple case series (for instance, occlusive disease in ~29 out of 60 treated HIV-positive patients in one large cohort). Spontaneous dissections appear less frequently in the literature, but dissection-associated pseudoaneurysms have been described and likely account for approximately 10-15% of documented structural vascular complications in HIV-positive patients [6]. Proposed mechanisms include chronic inflammation, immune activation, smooth muscle dysfunction, and elevated homocysteine levels due to nutritional deficiencies, all of which may compromise vascular integrity [7].

Spontaneous arterial dissections involving the cervical carotid and vertebral arteries have been reported in HIV‑positive individuals; however, isolated celiac artery involvement is exceedingly rare [8]. Cocaine, a potent sympathomimetic agent, is another significant contributor to arterial pathology. Acute cocaine use triggers abrupt surges in blood pressure and heart rate, induces intense vasoconstriction, and rapidly increases shear stress on the arterial wall, often precipitating intimal tears and arterial dissection within minutes to hours of use, particularly in younger individuals without pre‑existing vascular disease [9]. This contrasts with chronic use, which over time promotes cumulative vascular injury through smooth muscle apoptosis, extracellular matrix degeneration, endothelial dysfunction, and increased aortic stiffness-weakening arterial structural integrity and raising long‑term dissection risk [10].

Although vascular dissections in cocaine users remain relatively uncommon, they disproportionately affect younger individuals and often present dramatically. In the International Registry of Acute Aortic Dissection (IRAD), cocaine use was identified in approximately 1.8 % of all aortic dissection cases [11]. In a compiled analysis of 45 cocaine‑related aortic dissections, 75% were Stanford type A (involving the ascending aorta), the median time from last cocaine use to symptom onset was approximately one hour, and patients were predominantly young men (mean age ~41 years) [12].

Although few reports exist linking cocaine to visceral artery dissections, its pathophysiologic effects strongly support a causal role [13]. The coexistence of HIV infection and chronic cocaine abuse in our patient likely created a unique and high-risk vascular environment, predisposing him to spontaneous celiac artery dissection even in the absence of conventional risk factors. In this report, we describe a rare case of SICAD in an HIV-positive patient with recent cocaine use. We review the clinical presentation, imaging findings, as well as conservative management, and we highlight the potential mechanisms by which HIV and cocaine may have contributed to this rare event. This case underscores the importance of maintaining a high index of suspicion for visceral artery dissection in patients presenting with acute abdominal pain, even when atypical risk factors are present. 

## Case presentation

Our patient is a 45-year-old man with a history of HIV infection, diagnosed 10 years ago. He is currently on antiretroviral therapy (ART) with an undetectable viral load but also has a history of chronic cocaine abuse. He presented to the emergency department with severe upper abdominal pain. The pain began suddenly about one hour after the patient admitted to having snorted cocaine. He described the pain as a constant, “tearing” epigastric pain radiating to his back. He denied any recent trauma, vomiting, or melena. Past medical history was significant for HIV (CD4 count ~500 cells/µL on last check) and intermittent medication non-adherence, as well as untreated hyperlipidemia. He had no known history of hypertension or connective tissue disorders.

Intermittent ART adherence can contribute to vascular dysfunction in several ways. Periods of non-adherence are associated with transient increases in viral replication and systemic immune activation, which promote endothelial injury through pro-inflammatory cytokines and oxidative stress [1]. These fluctuations disrupt nitric oxide (NO) signaling, reduce endothelium-dependent vasodilation, and increase vascular stiffness [2,3]. Studies have shown that inconsistent ART use correlates with greater carotid intima-media thickness and impaired flow-mediated dilation when compared to patients with sustained viral suppression [4]. Furthermore, intermittent ART exposure may impair endothelial progenitor cell function, diminishing vascular repair capacity and contributing to long-term arterial injury [5].

On arrival to the emergency department, vital signs showed blood pressure 168/100 mmHg, heart rate 110/min, respiratory rate 20/min, and oxygen saturation 99% on room air. He appeared in distress due to pain. Cardiovascular examination revealed a regular tachycardic rhythm without murmurs. Abdominal exam found moderate epigastric tenderness but no guarding or rebound; there was no abdominal pulsatile mass and bowel sounds were normal.

Initial laboratory studies showed a mild leukocytosis of 12,000/µL and a lactic acid of 2.8 mmol/L. Cardiac enzymes were normal. Liver enzymes, pancreatic enzymes, and renal function were within normal limits. Given the patient’s risk factors and severe pain, an acute aortic syndrome or visceral ischemia was suspected. A 12-lead-electrocardiogram showed sinus rhythm with a rate of 110 beats per minute. Urine toxicology was positive for cocaine. Rapid HIV testing was positive, which was consistent with the patient’s known history. The emergency physician ordered an urgent contrast-enhanced CT scan of the chest and abdomen to evaluate for aortic dissection or mesenteric ischemia. Certain CT features may predict progression in isolated visceral artery dissections, including a patent or partially thrombosed false lumen, ulcer-like projections, longer dissection length, and morphologic types with persistent false lumen flow (e.g., Sakamoto types I and III) [5]. Key laboratory findings are summarized in Table 1.

**Table 1:** Lab results WBC: White blood cell count

Lab Test	Result	Reference Range	Comments
WBC	12,000 /µL	4,000–10,000 /µL	Mild leukocytosis (stress, pain)
Lactic acid	2.8 mmol/L	0.5–2.2 mmol/L	Mildly elevated (possible hypoperfusion)
Cardiac enzymes	Normal	-	No evidence of myocardial infarction
Liver enzymes	Within normal limits	-	-
Pancreatic enzymes	Within normal limits	-	-
Renal function	Within normal limits	-	-
HIV rapid test	Positive	Negative	Known HIV infection
Urine toxicology	Positive for cocaine	Negative	Consistent with reported use

**Figure 1:**
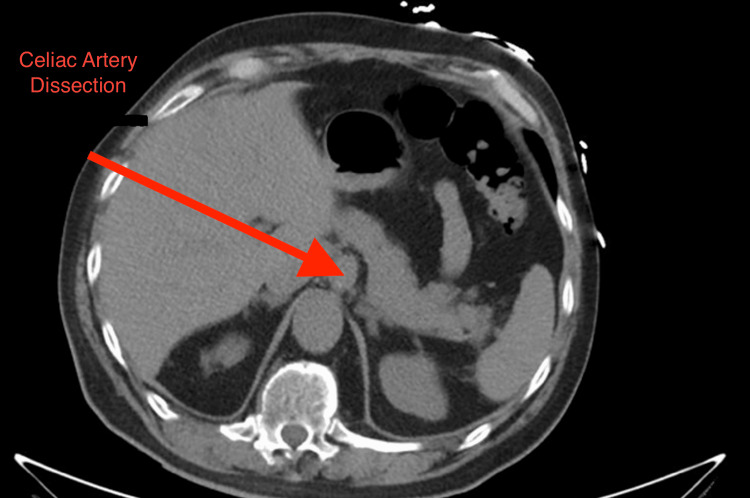
Contrast-enhanced axial CT abdomen The contrast-enhanced axial CT image of the abdomen demonstrating aneurysmal dilation of the celiac artery (arrow), consistent with focal dissection. The true lumen is narrowed, and there is no evidence of aortic involvement.

**Figure 2:**
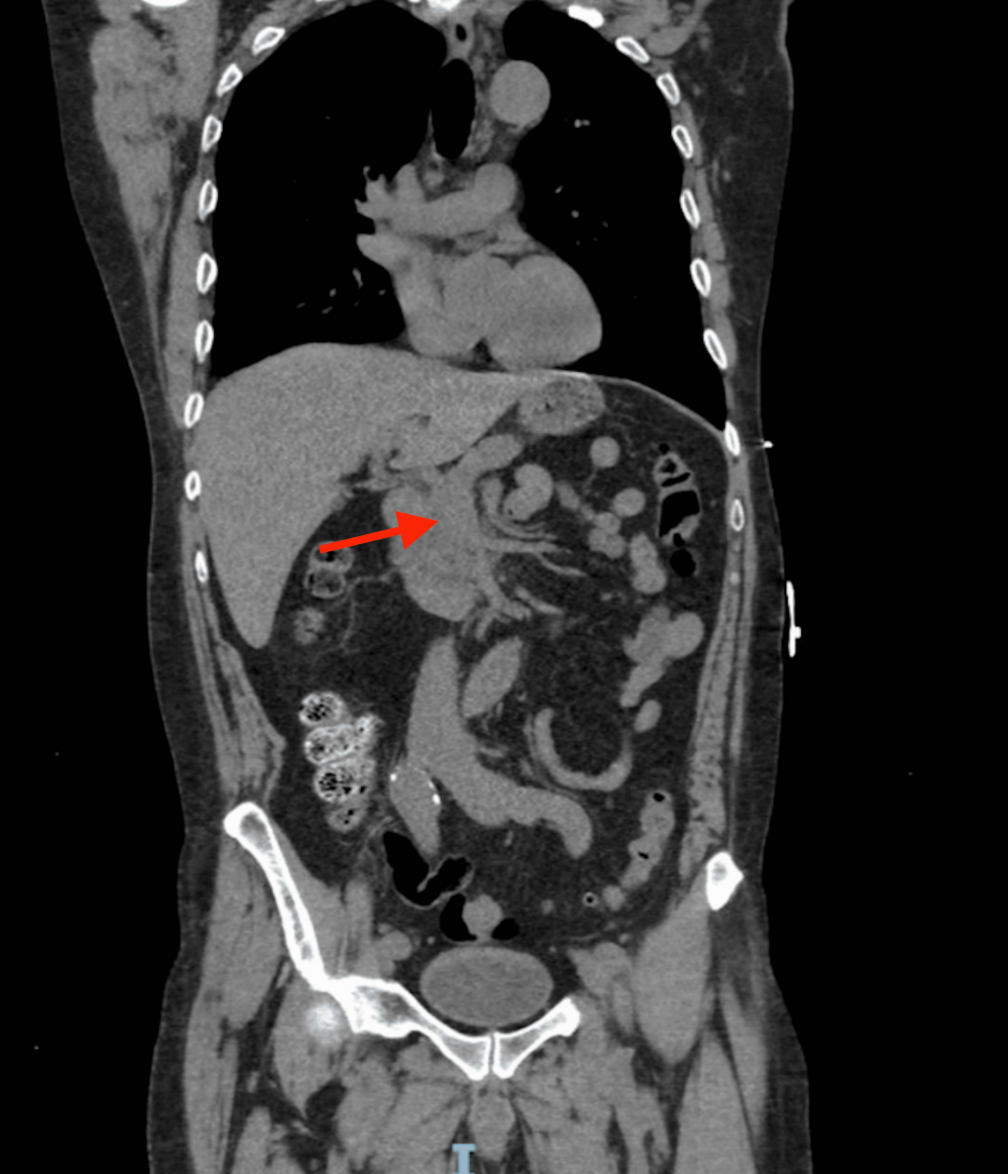
Coronal reformatted CT angiogram showing dissection of the proximal celiac artery This coronal reformatted CT angiogram shows dissection of the proximal celiac artery (arrow). The dissection flap is seen extending from the celiac origin without evidence of extension into the aorta or superior mesenteric artery. Surrounding organs are well perfused, with no signs of bowel ischemia or infarction.

The CT angiogram of the abdomen revealed a dissection flap in the celiac artery, beginning just distal to its origin from the aorta and extending into the proximal common hepatic artery. The true lumen of the celiac trunk was narrowed by an intimal flap, and there was a smaller false lumen with no evidence of active extravasation. The dissection did not involve the superior mesenteric artery or the aorta, and there were no signs of bowel ischemia or organ infarction. Radiology interpreted the findings as a SICAD (Stanford type not applicable since the aorta was spared). No aneurysmal dilation was noted at the celiac trunk, though a small perivascular hematoma was seen, suggesting a contained leak. Figure 1 shows the CT of our patient’s abdomen demonstrating aneurysmal dilation of the celiac artery which is consistent with focal dissection. Figure 2 is our patient’s CT angiogram showing dissection of the proximal celiac artery.

Our patient was admitted to the ICU for close monitoring. Both cardiology and vascular surgery consult services were involved early in the hospital course. Given that the patient was hemodynamically stable with no evidence of end-organ ischemia, all consultants agreed to pursue conservative management. The blood pressure was tightly controlled with intravenous esmolol infusion initially, targeting a systolic blood pressure < 120 mmHg to reduce shear stress on the arterial wall. Pain was managed with intravenous morphine, which also helped alleviate sympathetic surges. The patient was started on a heparin drip for anticoagulation, given the concern for potential thrombosis in the false lumen of the dissection; low-dose aspirin was also initiated for additional antiplatelet effect. Laboratory tests showed no coagulopathy or connective tissue disorder markers. The patient’s HIV regimen was continued during hospitalization, and his CD4 count on admission was 480 cells/µL with an HIV viral load < 50 copies/mL.

Over the first 48 hours of admission, the patient’s abdominal pain gradually improved to a mild ache. Blood pressure was transitioned from IV esmolol to oral metoprolol as he stabilized. Repeat abdominal exam remained benign, and lab markers of organ perfusion (lactic acid, liver enzymes) stayed normal. Vascular surgery recommended against any immediate invasive intervention since there were no signs of expanding hematoma or organ compromise. Cardiology monitored for any cardiac involvement; a transthoracic echocardiogram showed normal aortic root and ascending aorta with no dissection, and normal left ventricular function.

On hospital day 5, a follow-up CT angiography was performed to ensure the dissection had not progressed. The repeat imaging showed an unchanged dissection in the celiac artery, with intact flow to the hepatic and splenic arteries via the true lumen and well-developed collateral circulation. The presence of robust collateral perfusion is a favorable prognostic indicator in visceral artery dissection, as it reduces the risk of end-organ ischemia and supports conservative management by maintaining adequate perfusion despite true lumen compression. No new abnormalities were noted. Given the stable imaging and the patient’s clinical improvement, he was deemed safe for discharge. He was transitioned to oral antihypertensive therapy with metoprolol and lisinopril to maintain a target systolic blood pressure below 120 mmHg. He was prescribed beta-blockers to reduce his heart rate and limit shear stress on the arterial wall, and angiotensin-converting enzyme (ACE) inhibitors to improve endothelial function and reduce arterial wall tension. Together, they form the cornerstone of medical management in preventing dissection progression and recurrence [7]. The patient was continued on dual antithrombotic therapy with aspirin 81 mg daily and rivaroxaban 5 mg daily for a planned three-month course to facilitate false lumen thrombosis and minimize the risk of distal embolization. He was strongly advised to abstain from cocaine and tobacco and received counseling on the importance of strict adherence to his HIV treatment regimen and outpatient follow-up.

Given the high vascular risk associated with ongoing cocaine use, targeted substance use counseling was initiated during his admission. Evidence-based strategies shown to be effective in vascular patients include motivational interviewing to enhance readiness to change, cognitive behavioral therapy to address triggers and coping mechanisms, contingency management programs that use incentives for abstinence, and referral to specialized addiction medicine or integrated behavioral-cardiovascular programs. These multidisciplinary interventions have been shown to reduce cocaine use and improve both substance use and cardiovascular outcomes [13].

At discharge, arrangements were made for outpatient follow-up with vascular surgery in one month and again at three months with repeat imaging. The plan included a follow-up contrast-enhanced CT scan at three months to assess for healing or any development of a celiac artery aneurysm. The patient was also referred to a cardiac rehabilitation program for supervised exercise and to addiction counseling services for substance abuse cessation support. HIV care was to be continued with his infectious disease specialist. Multidisciplinary follow-up models that integrate vascular surgery, infectious disease, addiction medicine, and cardiology have shown improved care coordination and outcomes in complex vascular-HIV cases, particularly when managed through structured clinics or case-conference-based care teams.

## Discussion

SICAD is a rare vascular condition, with fewer than 200 cases reported in the literature to date [1,2]. It is increasingly diagnosed due to greater utilization of CT angiography in the evaluation of acute abdominal pain. While most patients with SICAD are middle-aged men with traditional vascular risk factors, our case illustrates an atypical presentation involving a patient with HIV infection and recent cocaine use, two non-traditional but synergistic risk factors [2].

Cocaine use is a well-documented cause of acute vascular pathology. It exerts its effects primarily through potent sympathetic stimulation, resulting in severe hypertension, arterial vasospasm, and endothelial injury, all of which can increase shear stress on vessel walls and precipitate intimal tears [7-9]. Cocaine has been associated with dissections in coronary, carotid, aortic, and mesenteric vessels [8]. In our case, the patient developed acute, tearing epigastric pain within one hour of cocaine use, and presented with elevated blood pressure, supporting a temporal and mechanistic relationship. Chronic cocaine use may also degrade vascular integrity over time by promoting smooth muscle apoptosis and accelerating medial degeneration [9].

HIV infection, particularly when longstanding or poorly controlled, is also associated with a vasculopathy characterized by endothelial dysfunction, chronic inflammation, and smooth muscle proliferation [4,5]. Even in patients with virologic suppression, prior episodes of viremia and intermittent medication adherence, as seen in our patient, may lead to cumulative vascular injury. HIV has been linked to spontaneous arterial dissections in the carotid and vertebral arteries, though to our knowledge, isolated celiac artery dissection in this context has been rarely reported [6].

The pathophysiology in our case likely involved chronic arterial weakening due to HIV-related inflammation, with an acute surge in hemodynamic stress from cocaine serving as the precipitating event. This interaction between chronic and acute insults highlights the importance of a comprehensive understanding of nontraditional risk factors in vascular emergencies.

Management of SICAD is guided by the patient's hemodynamic stability, symptoms, and the presence or absence of complications. There are no randomized trials or definitive guidelines due to the rarity of this condition, but most published cases support conservative treatment as the first-line approach in stable patients without ischemia [2,3]. Conservative management is typically favored in hemodynamically stable patients who lack evidence of end-organ ischemia, arterial rupture, or rapidly expanding aneurysm. Our patient met these criteria, with stable vital signs, preserved organ perfusion, and no signs of vascular compromise on imaging. He was treated conservatively with strict blood pressure control using intravenous beta-blockers, later transitioned to oral therapy. He also received anti-thrombotic therapy, including initial anticoagulation with heparin followed by rivaroxaban, along with low-dose aspirin to promote false lumen thrombosis and prevent embolization. Serial imaging confirmed a stable dissection with no evidence of progression or branch compromise.

This approach aligns with other cases in the literature [2,3,10]. In a review by Cavalcante et al., over 90% of patients with SICAD managed non-operatively experienced symptom improvement without disease progression [2]. Surgical or endovascular intervention is typically reserved for patients with ongoing ischemia, expanding pseudoaneurysm, or failure of medical therapy [3,11].

Follow-up care is essential in such cases. Risk factor modification was emphasized, including counseling for cocaine cessation, referral to addiction recovery services, and optimization of HIV treatment. He was initiated on statin therapy to reduce long-term vascular risk. Given the increased risk of recurrent vascular events in patients with HIV, lifelong cardiovascular surveillance is warranted.

In summary, this case demonstrates that celiac artery dissection can occur in patients without traditional cardiovascular risk factors, particularly when novel contributors like HIV and cocaine use are present. Prompt diagnosis and appropriate conservative management can yield favorable outcomes. Clinicians should maintain a high index of suspicion for visceral artery dissection in patients presenting with severe abdominal pain and atypical risk profiles. Although data are limited, the long-term prognosis of medically managed SICAD is generally favorable, with most patients showing stabilization or resolution on follow-up imaging [14]. In HIV-positive patients, outcomes remain similarly positive when viral suppression is maintained and cardiovascular risk factors, such as hypertension, substance use, and dyslipidemia, are adequately addressed.

## Conclusions

Celiac artery dissection is a rare cause of acute abdominal pain that warrants consideration in the differential diagnosis, especially in patients with risk factors or unusual presentations. This case underscores that even in an HIV-positive individual without classic cardiovascular risk factors, cocaine use can serve as a powerful precipitant of arterial dissection. The combination of chronic HIV-associated arterial changes and acute cocaine-induced hypertension likely contributed to the vascular injury in our patient. Prompt imaging with CT angiogram was essential to diagnosis, and interdisciplinary management allowed for a successful outcome with non-surgical therapy. Most isolated celiac artery dissections can be managed conservatively with careful monitoring, reserving invasive interventions for those with complications. Our patient remained asymptomatic with stable imaging at three months and is expected to have a favorable long-term prognosis with continued blood pressure control and abstinence from cocaine. Clinicians should counsel patients on aggressive risk factor modification, including abstinence from cocaine and adherence to ART, to prevent recurrence and improve vascular health.

This case is unique in demonstrating how non-traditional but synergistic risk factors (HIV and cocaine use) can result in a vascular emergency in a relatively young patient without underlying atherosclerotic disease. It highlights a potentially under-recognized intersection of risk factors that may justify earlier imaging or heightened clinical vigilance in HIV-positive patients presenting with acute abdominal pain and known substance use. From a public health and clinical education perspective, the case emphasizes the importance of integrating substance use screening and cardiovascular risk assessment into routine HIV care, particularly given the vascular complications associated with both chronic infection and stimulant abuse. In terms of future management, this case supports the value of multidisciplinary, non-surgical approaches in select patients and contributes to the growing body of evidence that may inform the development of formal diagnostic and therapeutic guidelines for visceral artery dissections, especially in atypical populations.
